# Supplementary material for: High-speed and energy-efficient non-volatile silicon photonic memory based on heterogeneously integrated memresonator
Source: Nat Commun. 2024 Jan 16;15:551. doi: 10.1038/s41467-024-44773-7 (PMC10791609; doi:10.1038/s41467-024-44773-7)
Supplement: Supplementary file 1 — Supplementary Information [file 41467_2024_44773_MOESM1_ESM.pdf]

Supplementary Information for

High-Speed and Energy-Efficient Non-Volatile Silicon Photonic Memory Based on Heterogeneously Integrated Memresonator

Bassem Tossoun *et al.*

*Corresponding author. Email: bassem.tossoun@hpe.com

This PDF file includes:

Supplementary Notes
Supplementary Figures
Supplementary References

Supplementary Notes

Supplementary Note 1. Switching Energy

The switching energy can then be calculated by integrating the product of $I_{memristor}$ x $V_{memristor}$ under the duration of the voltage pulse. In the case of the SET cycle, $V_{memristor} = V_{SET}$, the amplitude of the voltage pulse used to SET the memristor into the LRS, and $I_{memristor} = I_{SET}$, the maximum current in the memristor when the device switches to the LRS. In the case of the RESET cycle, $V_{memristor} = V_{RESET}$, the amplitude of the voltage pulse used to RESET the memristor into the LRS, and $I_{memristor} = I_{RESET}$, the peak absolute current before the current decreases due to switching. The current values are measured with a Keysight B1500A source measuring unit while pulses are sent to the device with the Keysight B1525A pulse generator unit. Supplementary Figure 1 shows the experimental set-up used to measure the device characteristics.

To take account of the variation of switching conditions across devices, we averaged the switching power across 4 devices, all having been switched using 4 V and 5 V, 100 ns voltage pulses and 5 ns rise and fall times. These values can also be extracted from the resistance measurements shown in Figure 4(d) in the manuscript. The average switching power for SET is the product of $I_{memristor}$ x $V_{memristor}$ = 100 μA x 5 V = 500 μW ± 80 μW and the average switching energy for SET is 500 μW x 300 ps = 0.15 pJ ± 0.03 pJ. The average switching power for RESET is 100 μA x 4 V = 400 μW ± 90 μW and the average switching energy for RESET is 400 μW x 900 ps = 0.36 pJ ± 0.08 pJ. In order to read the device, it costs less than 2.5 μA x 2 V = 5 μW of power and only 5 μW x 1 ns = 5 fJ of energy when reading in the LRS, and 1 nA x 2 V = 2 nW and only 2 nW x 1 ns = 2 aJ of energy to read in the HRS.

When used in practice, the memristor typically operates using voltage pulse sequences: a write followed by a read cycle, and an erase followed by a read cycle. One voltage pulse is used to switch the state and write/erase data onto the memristor, and another pulse is used to read the current in the memristor. In the case of the memristor, a read voltage is applied to read the resonant wavelength of the device. In between read and write cycles, there is zero static power consumption.

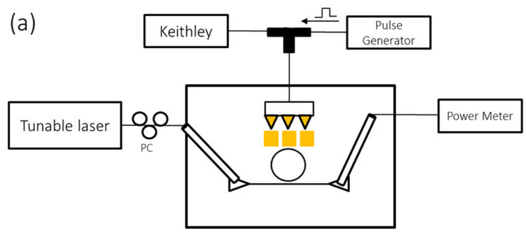

Supplementary Figure 1. Experimental Setup Schematic

(a) Schematic diagram of the experimental setup used during the endurance and retention time measurements. (b) Schematic diagram of the experimental setup used during the of the switching speed measurement.

Supplementary Note 2. Insertion and Coupling Loss

In order to calculate the coupling and insertion losses within the memresonator, we used the following equations [1]:

$$\mathcal{T} \equiv \left(\frac{t^2 + \alpha^2 - 2\alpha t \cos\phi}{1 + \alpha^2 t^2 - 2\alpha t \cos\phi}\right)$$

$$\mathcal{F} \equiv \frac{\Delta\lambda_{\text{FSR}}}{\Delta\lambda_{\text{FWHM}}}$$

$$\mathcal{E} \equiv \frac{T_{max}}{T_{min}}$$

$$\mathcal{E} = \left[\frac{(\alpha + t)}{(\alpha - t)}\frac{(1 - \alpha t)}{(1 + \alpha t)}\right]^2$$

$$\cos(\pi/\mathcal{F}) = \frac{2\alpha t}{1 + \alpha^2 t^2}$$

$$A \equiv \frac{\cos(\pi/\mathcal{F})}{1 + \sin(\pi/\mathcal{F})}$$

$$B \equiv 1 - \left[\frac{1 - \cos(\pi/\mathcal{F})}{1 + \cos(\pi/\mathcal{F})}\right]\frac{1}{\mathcal{E}}$$

$$(\alpha, t) = \left(\frac{A}{B}\right)^{1/2} \pm \left(\frac{A}{B} - A\right)^{1/2}$$

$\mathcal{F}$ is the finesse, $\mathcal{E}$ is the extinction ratio, α is the loss coefficient, or the optical loss in one roundtrip of the microring resonator, t is the coupling coefficient, FSR is the free spectral range of the microring resonator, and FWHM is the full-width at half maximum of the resonance. In our case the FSR was around 2.808 nm and the FWHM was around 0.13 nm, giving a finesse of around 21.5. The Q-factor of the ring is around 9,933 and the extinction ratio is 27.55.

A Lorentzian fit was used to curve fit the resonances for extraction of the coefficients and the resonance wavelength throughout measurements made. The function used is shown below:

$$f(\lambda) = A_{\text{res}}\left(1 - \left(1 - \frac{1}{rE}\right)\frac{(1/2(\text{FWHM}))^2}{(\lambda - \lambda_0)^2 + (1/2(\text{FWHM}))^2}\right)$$

where A_{res} is the normalization factor of the resonance, rE is the extinction coefficient, FWHM is the full width at half maximum, and λ_0 is the mean wavelength of the Lorentzian fit. Fig. S3 displays a plot of a resonance including the Lorentzian fit.

Fig. S4 are plots including the different memresonator structures that we measured and their corresponding coupling and insertion losses. With α = 0.945, we can calculate the insertion loss in the HRS while the device is being read through the follow equation:

$IL = 10 * log_{10}\alpha$ = 0.047 dB

In the LRS, α = 0.937, we can calculate the insertion loss to be while the device is being read:

$IL = 10 * log_{10}\alpha$ = 0.048 dB

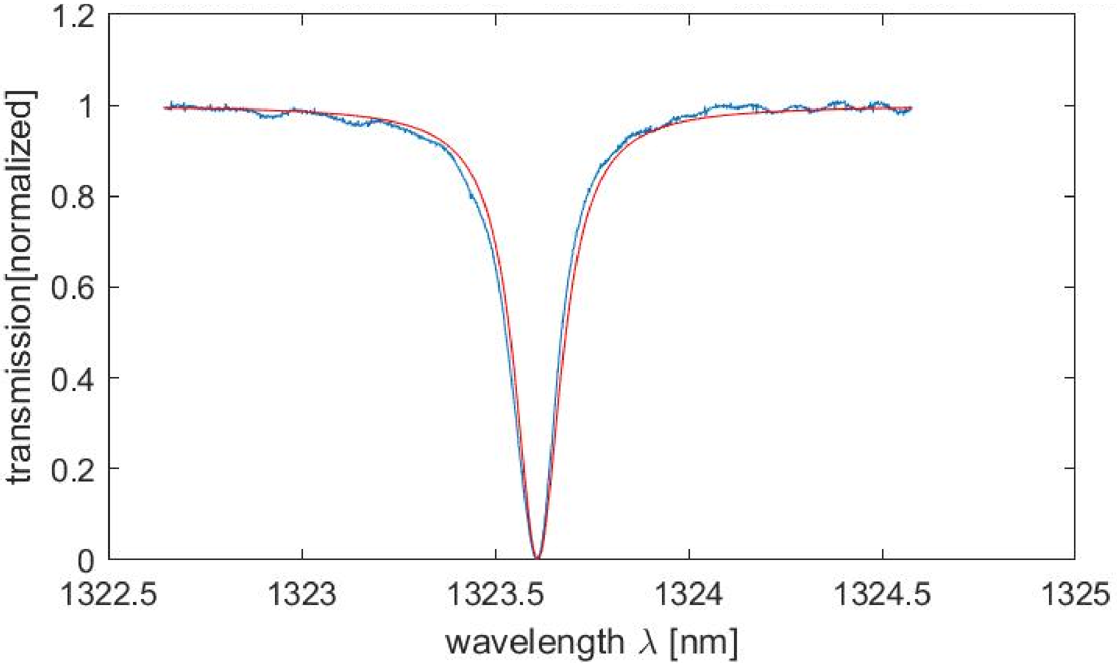

Supplementary Figure 2. Transmission Spectrum of Memresonator with Lorentzian Fit
Transmission spectrum for a resonance of the memresonator including the Lorenztian fitted curve.

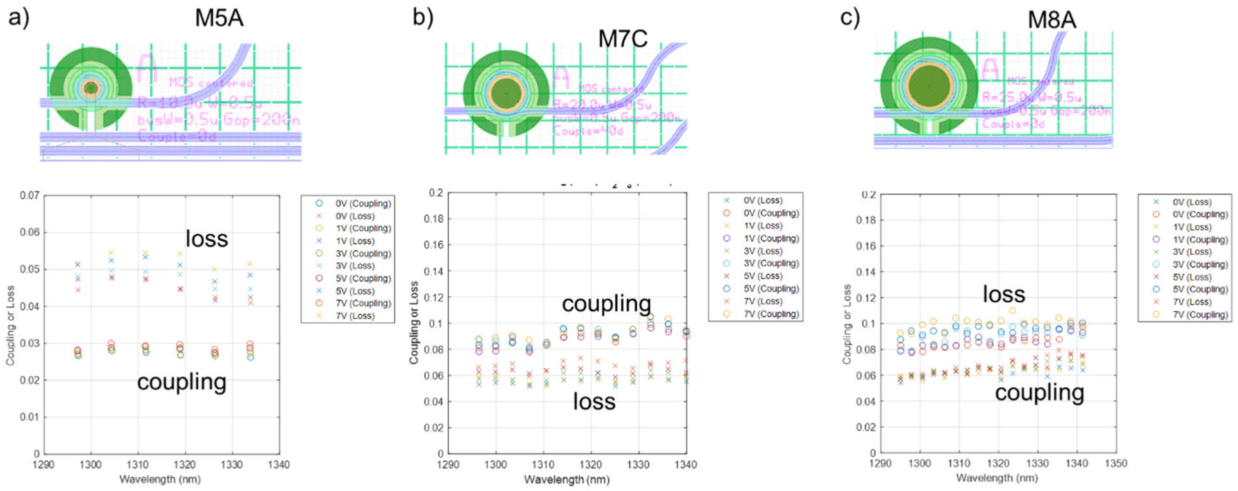

Supplementary Figure 3. Insertion Loss and Coupling Loss of Memresonator

a) Insertion and coupling loss measured at varying applied bias voltages for a 10 μm radius memresonator, b) a 20 μm radius memresonator, c) and a 25 μm radius memresonator.

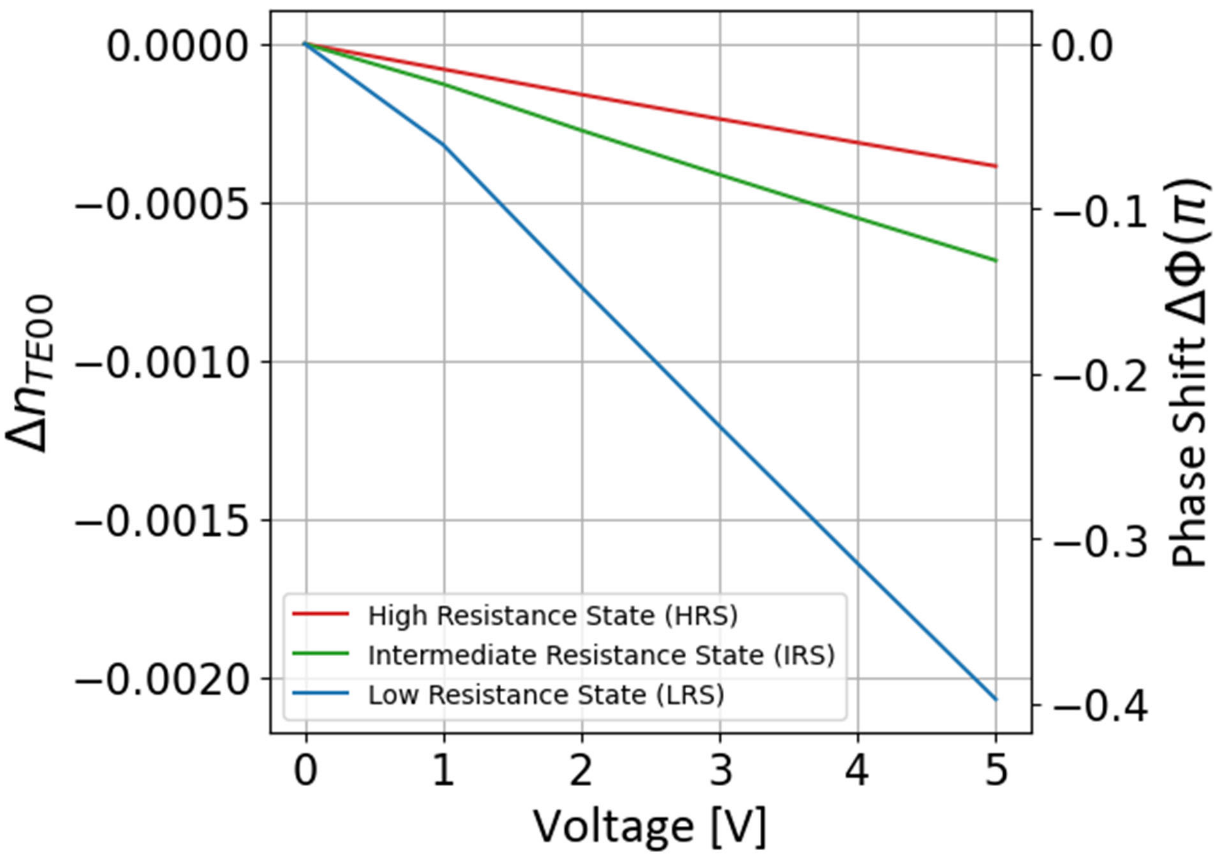

Supplementary Figure 4. Index Change and Phase Shift Versus Bias Voltage
Effective refractive index change and phase shift as a function of bias voltage of the memresonator.

Supplementary Note 3. Leakage Current

The leakage current of the memristor was measured in the high resistance state (HRS) and displayed in Supplementary Figure 5 using a voltage sweep from 0 to 5 V.

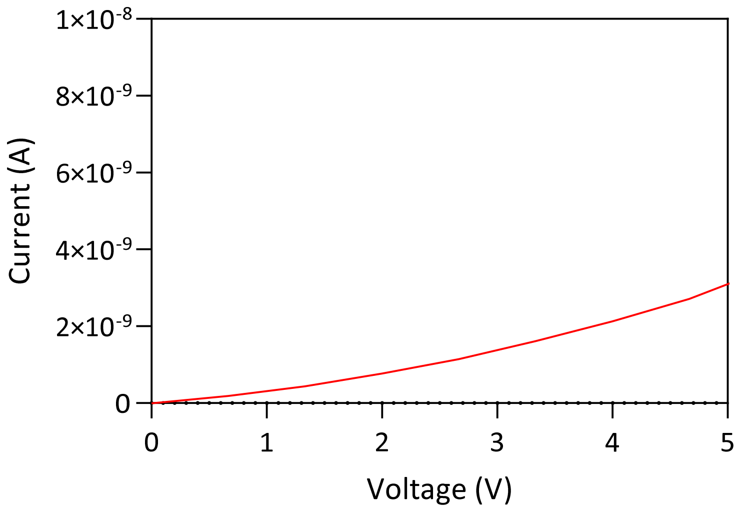

Supplementary Figure 5. Leakage Current
Leakage current of the memristor in the HRS.

Supplementary Note 4. Broadband Spectrum

We measured the broadband spectrum of the memresonator without extracting any losses from the grating couplers or the waveguide losses and plotted it in Supplementary Figure 6.

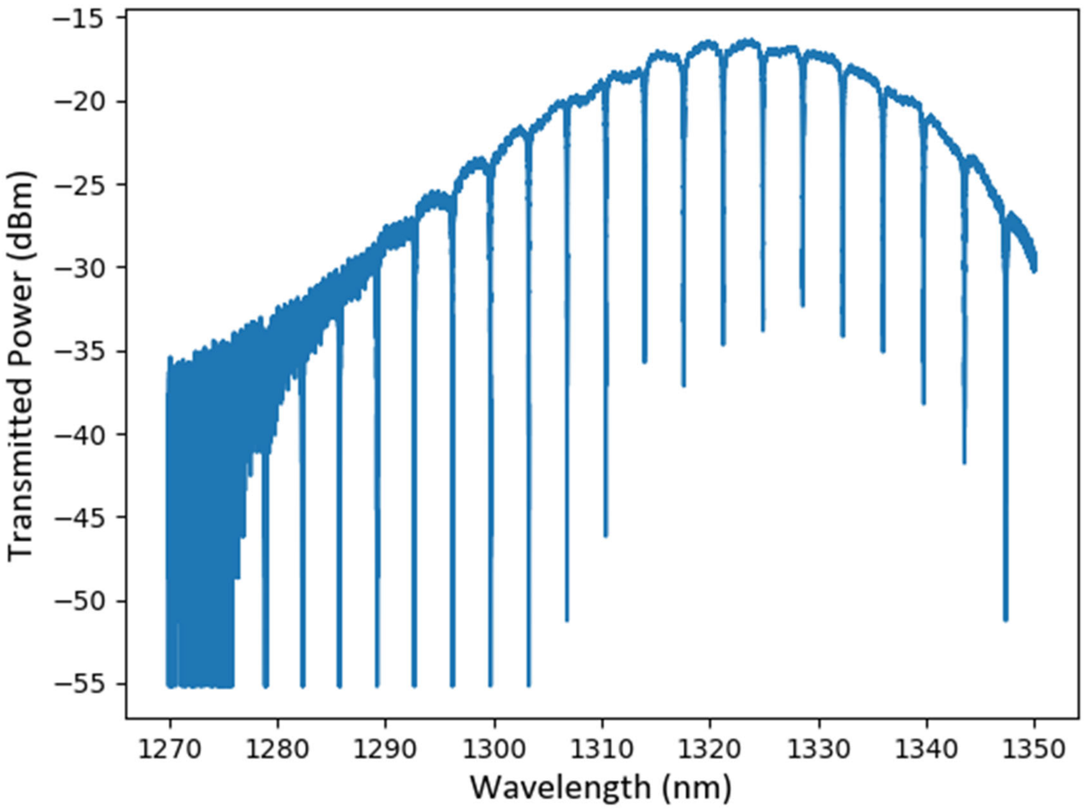

Supplementary Figure 6. Broadband Spectrum
Broadband spectrum of the memresonator without extracting losses from the grating coupler.

Supplementary Note 5. Phase Shift Calculation

In order to calculate the phase shift of the memresonator, we used the following formula:

$$Phase\ shift = \frac{2\pi\Delta\lambda}{\mathrm{FSR}} = \frac{2\pi * 0.08\ \mathrm{nm}}{2.808\ nm} \sim 0.18\pi$$

where FSR = 2.808nm, and the $\Delta\lambda\ =\ 0.08$ nm between the LRS and the HRS at a read voltage of 2 V. This gives us an estimated L_π of $(\pi/0.18\pi)$ * 2*π*10μm = 0.349 mm $\approx 0.35\ mm$.

Supplementary Note 6. Thermal simulations

A 15 nm × 15 nm × 20 nm filament of aluminum was inserted in the Al_2O_3 layer to act as a conductive path for current to flow in the charge, heat, and optical simulations. The simulations were performed using Ansys Lumerical Finite Element IDE and Finite Difference IDE. The dimensions of the aluminum conductive filament were chosen to replicate conductive filaments formed in similar devices [2, 3, 4]. The charge simulations were done first to get the carrier concentration within the waveguide. The charge simulation data was then imported into the Finite Difference IDE solver to calculate the effective index of refraction for the TE modes in the waveguide. Thermal simulations were also done in Finite Element IDE and also imported into the Finite Difference IDE solver in the calculations of the effective index of refraction.

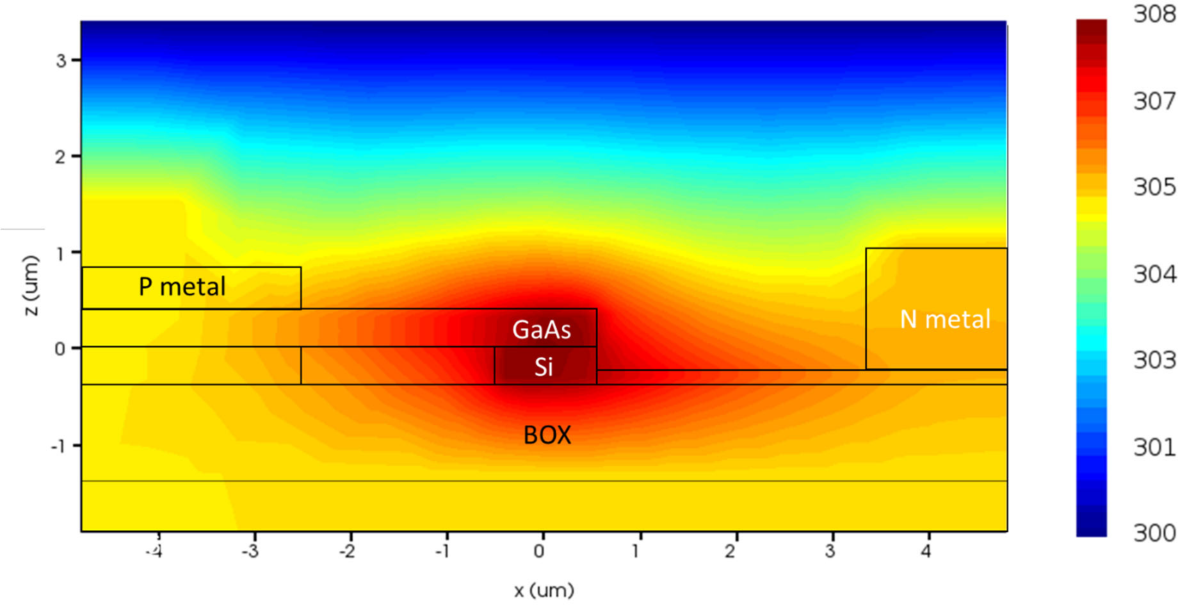

Supplementary Figure 7. Thermal Simulations

Thermal simulations of memristive III-V/Si waveguide in the LRS and 10 V applied. Localized Joule heating occurs within the CF located inside of the interfacial oxide layer between the III-V and Si.

Supplementary Note 7. Charge Simulations

Charge simulations were used to determine the carrier concentration as a function of the memristor state and location within the device, as well as corresponding band diagrams, can be found in Supplementary Figure 8. Due to different doping levels in the GaAs and Si layers, free carriers around the Al_2O_3 oxide layer are depleted at zero bias. Without a bias voltage applied on the memristor, the doped p-Si is largely depleted because of the doped n-GaAs on the lower side of the waveguide, introducing negligible background free carrier absorption (FCA) in p-Si. Once a forward bias is applied to the memristor when in the HRS, free holes and electrons quickly accumulate in the p-Si and n-GaAs, respectively, and their concentrations increase when approaching the oxide/semiconductor interface. Electrons accumulate on the interface of the Al_2O_3 layer and the n-GaAs layer, and holes accumulate at the interface of the p-Si and the Al_2O_3 layer while the memristor is the HRS.

The charge density change for electrons and holes in the HRS can be described as [5]:

$$\Delta N_e = \Delta N_h = \frac{\varepsilon_0 \varepsilon_r}{q t_{ox} t}[V - V_{FB}] \quad (1)$$

where ΔN_e is the change in electron concentration, ΔN_h is the change in hole concentration, ε_0 is the vacuum permittivity, q is the elementary charge, ε_r is the relative permittivity of the oxide layer, t_{ox} is the thickness of the oxide layer, t is the effective charge layer thickness, V is the applied bias voltage, and V_{FB} is the flat-band voltage of the memristor in the HRS. The effective index of refraction in the waveguide decreases due to the plasma dispersion effect in the Si and GaAs layers of the waveguide and the output wavelength is reduced as a result [6]. The change in the effective index of refraction of the Silicon portion of the waveguide, Δn_{eff_Si}, in the HRS can be described by the Drude-Lorentz model below [7]:

$$\Delta n_{eff\_Si} = -\frac{q^2 \lambda^2}{8 \pi^2 c^2 \varepsilon_0 n}\left(\frac{\Delta N_e}{m_e} + \frac{\Delta N_h}{m_h}\right) \quad (2)$$

where λ is the optical wavelength, c is the velocity of light in vacuum, n is the refractive index of the material unperturbed, m_e is the effective mass of electrons in the material, m_h is the effective mass of holes in the material.

The change in the effective index of refraction of the Gallium Arsenide portion of the waveguide, Δn_{eff_GaAs}, in the HRS can be described by the formula:

$$\Delta\, n_{eff\_GaAs} = -\frac{q^2\,\lambda^2}{8\,\pi^2\,c^2\,\varepsilon_0\,n}\left(\frac{\Delta\,N_e}{m_e}+\frac{\Delta\,N_h}{m_h}\right)+\frac{2ch}{q^2}P\int_0^{\infty}\frac{\Delta\alpha(\Delta\,N_e,\Delta\,N_h,E')}{E'^2-E^2}dE' \qquad (3)$$

where h is Planck's constant, E is the photon energy, and P indicates the principal value of the integral, and $\Delta\alpha$ is the bandfilling-induced change in absorption defined as

$$\begin{aligned}\Delta\alpha(\Delta\,N_e,\Delta\,N_h,E) &= -\Big(\frac{C_{hh}}{E}\sqrt{E-E_g}[f_{t'}(E_{ah})-f_c(E_{bh})-1] \\ &\quad+\frac{C_{lh}}{E}\sqrt{E-E_g}[f_{t'}(E_{al})-f_c(E_{bl})-1]\Big)\end{aligned} \qquad (4)$$

where C_{hh} and C_{lh} are constants involving materials parameters for heavy and light holes, $f_c(E_b)$ is the probability of a conduction band state of energy E_b being occupied by an electron, and f_v (E_a) is the probability of a valence band state of energy E_a being occupied by an electron and are given by the Fermi-Dirac distribution functions [8]. We are assuming that the change in effective index of refraction due to bandgap shrinkage is negligible. Subsequently, this change in effective index of refraction causes a change in the resonant wavelength of the MRR [9]:

$$\Delta\lambda_r = \frac{(\Gamma_{Si}\Delta n_{eff\_Si}+\Gamma_{GaAs}\Delta n_{eff\_GaAs})\lambda_r}{n_g} \qquad (5)$$

where Γ_{Si} is the optical confinement factor of the fundamental mode in the Silicon portion of the waveguide, Γ_{GaAs} is the optical confinement factor of the fundamental mode in the Gallium Arsenide portion of the waveguide, Δn_{eff} is the change in the effective index of the waveguide at the resonant wavelength, λ_r, and n_g is the group index of the hybrid III-V/Si waveguide. Using a III-V material warrants a significant increase in the refractive index of the waveguide due to its

high carrier mobility. By using a thinner oxide with a higher dielectric constant, such as HfO_2 or TiO_2, one could achieve a further increase in the plasma dispersion effect and tuning range [10].

Simulations show that as the carrier density within the waveguide increases, the plasma dispersion effect is enhanced and the blue shifting in the resonant wavelength occurs due to model refractive index decrease. This effect has been successfully used for energy-efficient optical phase tuning and high-speed optical modulation [11]. Thermal simulations also show that with a high enough electric field, current flow through the CFs increases and catalyzes localized Joule heating (see Supplementary Note 6). This effect increases the cavity modal refractive index and counteracts the plasma dispersion effect, eventually leading to a red shift in the resonant wavelength. It is worth noting that this effect is negligible under small read voltages and that the red shifting is only observed at high read voltages (>10 V).

Furthermore, the total change in the refractive index in the IRS or LRS can be modeled using the following formula [12]:

$$\Delta \lambda_r = -\frac{\lambda_r \Gamma}{n_g}\left(n_f \Delta N - \frac{dn_g}{dT} k_{th} R \cdot I^2\right) \quad (6)$$

where Γ is the optical confinement factor of the fundamental mode in the waveguide, n_g is the group index of the waveguide, n_f is the ratio between the change of silicon index and the change of carrier concentration when, ΔN is the change in the carrier concentration in the waveguide, dn_g is the thermo-optic coefficient, k_{th} is the thermal impedance of the memristor, R is the total effective series resistance, and I is the current flowing through the device.

$$\Delta N = \Delta N_p + \Delta N_i + \Delta N_n = \left(\frac{{n_i}^2}{N_D} + \frac{{n_i}^2}{N_A}\right)\left(e^{qV/kT} - 1\right) + \frac{IA\tau}{qt_{ox}} \quad (7)$$

The total change in the excess carrier concentration inside of the waveguide in the LRS, ΔN, can be calculated by summing the average excess carrier density inside the CF under high current level injection due to excess recombination, ΔN_n, the excess electron density inside of the p-Si region, ΔN_p, and the excess hole density in the n-GaAs region, ΔN_n, as according to Shockley's "Law of the Junction" [13, 14]. n_i is the intrinsic carrier concentration, N_D is the donor concentration, q is the charge of an electron, T is the device temperature, N_A is the acceptor concentration, τ is the carrier recombination lifetime inside of the CF and A is the area of the CF.

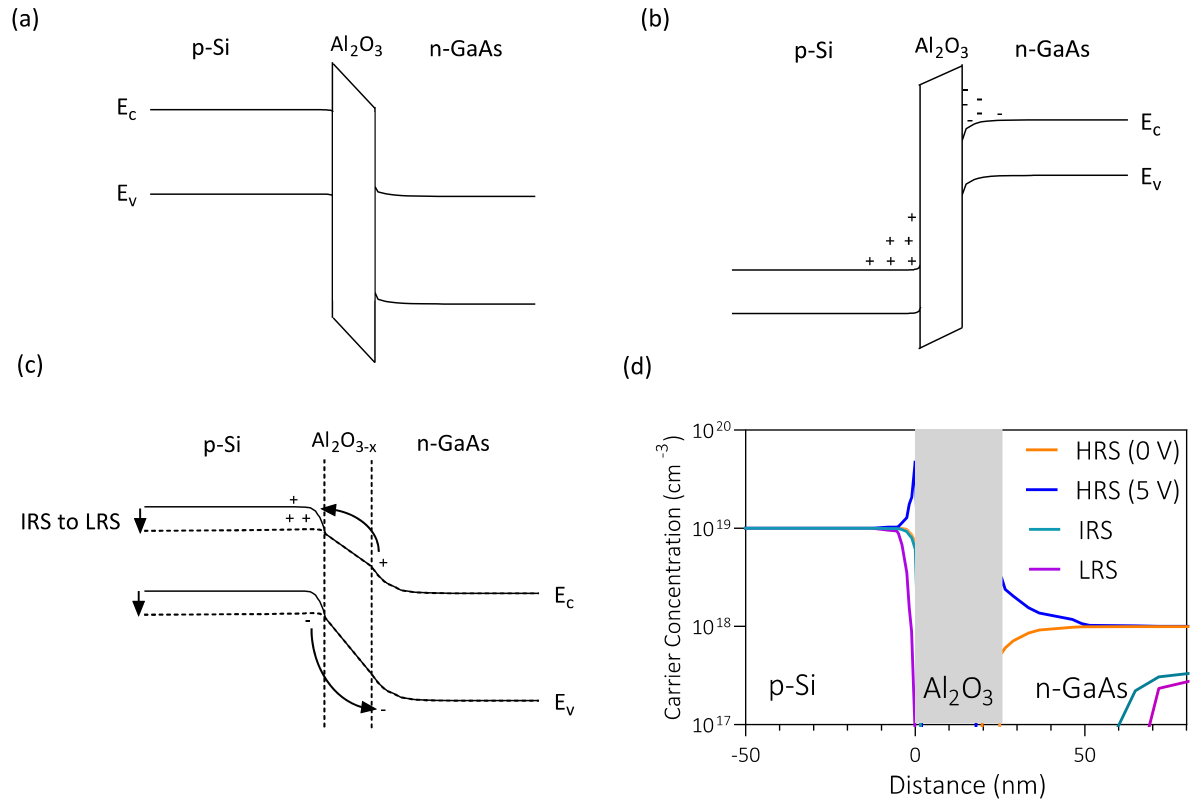

Supplementary Figure 8. Band diagrams and charge simulations
Band diagrams and charge concentration simulations of GaAs-Al_2O_3-Si memristor. (a) Band diagram of memristor in the HRS with zero applied bias voltage. (b) Band diagram of the memristor in the HRS with 5 V applied bias voltage. (c) Band diagram of the memristor in the LRS. (d) Simulated net carrier concentration in the memristor as a function of position in the device and bias voltage.

Supplementary Text References

[1] McKinnon, W. R., D-X. Xu, C. Storey, E. Post, A. Densmore, A. Delâge, P. Waldron, J. H. Schmid, and S. Janz. "Extracting coupling and loss coefficients from a ring resonator," *Optics Express*, vol. 17, no. 21 pp. 18971-18982, 2009.
[2] W. Sun, "Understanding memristive switching via in situ characterization and device modeling," *Nature Communications,* vol. 10, p. 13, 2019.
[3] X. Zhang, L. Xu, H. Zhang, J. Liu, D. Tan, L. Chen, Z. Ma and W. Li, "Effect of Joule Heating on Resistive Switching Characteristic in AlOx Cells Made by Thermal Oxidation Formation," *Nanoscale Research Letters,* vol. 15, p. 11, January 2020.
[4] Y. Wu, S. Yu, B. Lee and P. Wong, "Low-power TiN/Al_2O_3/Pt resistive switching device with sub-20 μA switching current and gradual resistance modulation," Journal of Applied Physics, vol. 110, p. 094104, November 2011.
[5] A. Liu, R. Jones, L. Liao, D. Samara-Rubio, D. Rubin, O. Cohen, R. Nicolaescu and M. Paniccia, "A high-speed silicon optical modulator based on a metal–oxide– semiconductor capacitor," vol. 427, p. 4, 2004.
[6] D. Liang, X. Huang, G. Kurczveil, M. Fiorentino and R. G. Beausoleil, "Integrated finely tunable microring laser on silicon," Nature Photonics, vol. 10, p. 719–722, November 2016.
[7] R. Soref and B. Bennett, "Electrooptical effects in silicon," IEEE Journal of Quantum Electronics, vol. 23, p. 123–129, January 1987.
[8] B. S. R. a. D. A. J. Bennett, "Carrier-induced change in refractive index of InP, GaAs and InGaAsP," IEEE Journal of Quantum Electronics, vol. 26, no. 1, pp. 113-122, 1990.
[9] W. Bogaerts, P. D. Heyn, T. Van Vaerenbergh, K. D. Vos, S. Kumar, T. Claes, P. Dumon, P. Bienstman, D. V. Thourhout and R. Baets, "Silicon microring resonators," p. 28, 2011.
[10] X. Huang, D. Liang, C. Zhang, G. Kurczveil, X. Li, J. Zhang, M. Fiorentino and R. Beausoleil, "Heterogeneous MOS microring resonators," in 2017 IEEE Photonics Conference (IPC), 2017.
[11] T. Hiraki, T. Aihara, K. Hasebe, K. Takeda, T. Fujii, T. Kakitsuka, T. Tsuchizawa, H. Fukuda and S. Matsuo, "Heterogeneously integrated III–V/Si MOS capacitor Mach–Zehnder modulator," Nature Photonics, vol. 11, p. 482–485, August 2017.
[12] R. Wu, C.-H. Chen, J.-M. Fedeli, M. Fournier, K.-T. Cheng and R. G. Beausoleil, "Compact models for carrier-injection silicon microring modulators," p. 10, 2015.
[13] A. Herlet, "The forward characteristic of silicon power rectifiers at high current densities," Solid-State Electronics, vol. 11, p. 717–742, August 1968.
[14] W. Shockley, "The theory of p-n junctions in semiconductors and p-n junction transistors," The Bell System Technical Journal, vol. 28, p. 435–489, July 1949.
